# Isobutanol tolerance in *Ralstoniaeutropha*

**DOI:** 10.1186/1753-6561-8-S4-P232

**Published:** 2014-10-01

**Authors:** Amanda Bernardi, Cláudia Gai, Jingnan Lu, Christopher Brigham, Anthony Sinskey

**Affiliations:** 1 Department of Biology, Massachusetts Institute of Technology, 77 Massachusetts Avenue, Cambridge, MA 02139, USA; 2 Universidade Federal de Alfenas, MG, Brazil; 3 Department of Chemistry, Massachusetts Institute of Technology, 77 Massachusetts Avenue, Cambridge, MA 02139, USA; 4 Department of Bioengineering, University of Massachusetts Dartmouth, 285 Old Westport Road, North Dartmouth, MA 02747, USA; 5 Department of Biology, Division of Health Sciences and Technology, Engineering Systems Division, Massachusetts Institute of Technology, 77 Massachusetts Avenue, Cambridge, MA 02139, USA

## Background

*Ralstonia eutropha *is bacterium known to naturally produce polyhydroxybutyrate (PHB) as carbon storage during nutrient starvation. Previously studies [1] showed that it is possible through the incorporation of an engineered biosynthetic pathway, to redirect carbon flux from PHB to the production of Isobutanol (IBT), a biofuel largely studied to replace the current fossil fuels in existing automobile engines. However *R. eutropha*, is unable to grow in the presence of IBT at concentrations above 0.2% (v v^-1^) which decreases its potential for industrial scale production. In order to minimize toxicity to the cells, we studied IBT tolerance to develop an IBT tolerant strain. We selected tolerant strains through experimental evolution and we confirmed the existence of mutations in 2 genes of *R. eutropha *evolved strains, the homologues of *acrA *and *acrA6 *in *Escherichia coli *, which were previously described as being related to IBT tolerance [2,3]. Those 2 genes were deleted from the wild type and engineered IBT-producing strains in order to evaluate improvement in IBT tolerance.

## Methods

*R. eutropha *strains were cultivated at 30ºC in rich and minimal media using fructose or IBT as carbon source. Experimental evolution was performed by sequential transfer method, adapted from previously work in alcohol tolerance [2,3]. For the deletion of the target genes, standard procedures were used as previously described [4] and all plasmids used for gene knock-out were constructed using Gibson Assembly technique. Isobutanol tolerance was compared by growth of strains in different IBT concentration, and by survival colony forming units (CFU ml^-1^) counting after exposition to elevated IBT concentration. Isobutanol consumption was tested by growth using IBT as exclusive carbon source. Culture aliquots were taken at different time points and IBT was extracted in order to evaluate IBT production.

## Results and conclusions

The effects of deletions were assessed separately and simultaneously, through IBT tolerance, consumption and production assays which showed that deletions did not significantly improve growth in the presence of IBT; however they increased the survival number of CFU ml^-1 ^in the presence of elevated concentrations of IBT. Moreover, an IBT producer strain with the *acrA *deletion was able to produce 3 times more IBT than its parental strain, which could potentially be associated with enhanced survival rate at high IBT concentration. Studies showed that the presence of extracellular IBT causes quinone depletion, reducing activity for enzymes that utilize it for their electron-carrier capability [5]. In *E. coli*, deletion of AcrAB-TolC units increased IBT tolerance by reducing this quinone depletion [3]. Our study has shown that this mechanism might be similar in *R. eutropha *response to IBT stress.
